# Sonographers’ perspectives towards the increase in medico-legal litigation: An exploratory qualitative study

**DOI:** 10.1177/1742271X261442776

**Published:** 2026-05-27

**Authors:** Megan Watkin-Bennett, Helen P White

**Affiliations:** 1 Medical Ultrasound Student, Birmingham City University, Birmingham, UK; 2 University of the West of England, Bristol, UK and Clinical Applications Specialist, Philips Electronics UK Ltd., south-west England and south Wales, UK; 3 Birmingham City University, Birmingham, UK

**Keywords:** sonographer burnout, litigation, second victim phenomenon, just culture, defensive practice

## Abstract

**Introduction:**

Medico-legal litigation is increasing within sonography, a profession already affected by high levels of occupational burnout and ongoing recruitment and retention challenges. Despite this trend, little research has explored how the possibility of litigation affects sonographers’ well-being or influences their professional practice.

**Method:**

This qualitative descriptive study was conducted in the United Kingdom. Purposive voluntary sampling recruited six National Health Service sonographers via social media. Online semi-structured interviews explored their perspectives on rising medico-legal litigation. Data were analysed using reflexive thematic analysis.

**Results:**

Four themes were identified: (1) The emotional and psychological burden, (2) The impact on sonography practices, (3) Work-related pressures, and (4) Supporting the sonographer. Litigation fear affected well-being and contributed to defensive behaviours, particularly in report writing. Participants also described systemic pressures, such as workload demands, limited appointment times, and misunderstandings about the sonographer’s role that increased the perceived risk of error and potential litigation. While transparency and professional accountability was valued, many highlighted the need for clearer guidance, learning opportunities, and stronger organisational support.

**Conclusion:**

Sonographers reported fear and uncertainty surrounding medico-legal litigation, alongside pressures that shaped both practice and emotional well-being. Although these concerns were prominent, participants also recognised the importance of transparency and accountability when supported by fair processes. Enhancing guidance, training, and organisational support may help reduce litigation fear and contribute to a more sustainable sonography workforce.

## Introduction

The UK sonography workforce faces significant occupational burnout, leading to emotional exhaustion and disengagement, which negatively impacts patient care quality and recruitment and retention (R&R) efforts.^1–4^ Increasing medico-legal litigation, driven by greater imaging use, technological advancements, extended practices, and rising patient expectations, is likely to worsen this burnout.^5,6^ Emerging medico-legal trends across Australasia have prompted the British Medical Ultrasound Society and the Australian Sonographers Association to issue joint guidance aimed at reducing litigation risks.^7^

Tracking sonographer-specific litigation is not possible due to the lack of direct regulation. Therefore, figures should be viewed as indicative rather than definitive. In 2006/2007, National Health Service Resolution (NHS-R) received 86 clinical negligence claims where the alleged harm occurred in radiology alone; by 2024/2025, this had risen to 523^8^ – an increase of 608%. However, they resolved the highest number of claims without court proceedings in 2024/2025.^9^ Yet, the average settlement time in radiology rose from 1.2 years in 2006/2007 to 1.7 years in 2024/2025.^10^

Concerns reported to the Health and Care Professions Council (HCPC) have risen as well. In 2023/2024, 0.36% of radiographer registrants were subject to fitness to practise concerns, up from 0.09% in 2004/2005.^11,12^ While these percentages remain low, the number of concerns has quadrupled. The Nursing and Midwifery Council (NMC) saw a similar trend; in the past 5 years, the highest number of concerns reported in a single month were seen in September 2024.^13^ The NMC also failed to meet the Professional Standards Authority’s (PSA) ‘Standards of Good Regulation’ again, only resolving on average 68.4% of cases within 15 months.^13^ In addition, five registrants under investigation died by suicide in 2023/2024.^14^ Although this represents less than 0.1% of those under investigation, it remains a deeply troubling figure.

Patients and families are the primary victims of potential clinical negligence claims but the impact on clinicians, often referred to as the second victim phenomenon (SVP), has only recently gained attention.^15^ A significant amount of international research has been comprised towards medical practitioners which often conclude that the psychological effects of litigation can be severe, including post-traumatic stress disorder.^16^ More commonly, guilt, shame, frustration, and fear, especially in blame-oriented environments is reported.^15,17^

NHS England promote a ‘just culture’ focused on learning rather than blame,^18^ yet systematic concerns remain. The House of Commons’ Health and Social Care Committee stated^19^ (p.55) “The system for compensating injured patients in England is not fit for purpose. It is grossly expensive, adversarial and promotes individual blame instead of collective learning”.

Although few claims reach court, complaint and claim rates continue to rise.^7^ SVP is said to contribute to burnout and early career exit, and litigation fear is reported to drive defensive practice, leading to unnecessary tests, patient anxiety, and system strain.^16^ Organisational support is considered vital, yet its effectiveness is poorly understood.^15^ Existing research focuses largely on medical practitioners, likely because they undertake higher-risk procedures and are more frequently involved in claims. In contrast, there is limited literature on sonographers, and what exists is either outdated or non-UK specific.

This study explores sonographers’ perspectives of litigation fear and how it influences well-being or professional practices. Findings aim to inform strategies that strengthen well-being, enhance support structures, and promote sustainable sonography practice.

## Methodology

**Figure 1.**
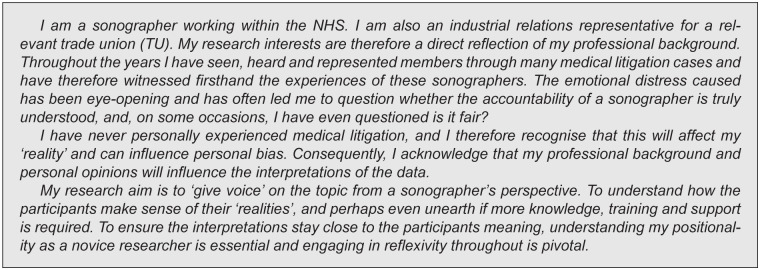
Positionality statement.

A qualitative descriptive study was utilised to align with the researcher’s critical realist perspective and to facilitate exploration of an under-researched topic. Semi-structured interviews were conducted by the researcher, who is an experienced sonographer, and the data were also analysed by the researcher using reflexive thematic analysis (RTA). To help address any quality concerns, a positionality statement (Figure 1) and an abstract of reflexivity were provided (Figure 3).^20^

A research proposal was reviewed by Birmingham City University’s (BCU) Health, Education and Life Sciences Faculty Academic Ethics Committee in March 2024 and after some minor alternations approved in May 2024. All participants were sent participant information, which outlined the study and included rights to confidentiality, safeguarding, data protection and withdrawal. An opportunity to ask questions was provided and written consent to use information gained during the research was obtained via the participant consent form. A debrief sheet was also provided which included links to sources of support.

Purposive voluntary sampling was used to recruit participants–since ‘sonographer’ is not a protected title, the criterion provided a clear, study-specific definition of the term. An initial target of six participants was established to facilitate data sufficiency rather than data saturation; provisions were made for further recruitment should analysis indicate the need for additional exploration to achieve a comprehensive understanding of the research question (RQ).

Recruitment took place between June and September 2024. An advertisement was posted on the researcher’s personal social media accounts (Facebook and LinkedIn). Interested individuals could click a link or scan a QR code, which directed them to a survey hosted by Jisc^®^ to assess whether individuals met the inclusion and exclusion criteria (Table 1). Ten eligible participants completed the survey. However, two participants did not provide email addresses, which prevented the researcher from contacting them further. In addition, one participant did not return the consent form; they were recontacted after 7 days, but after a further 7 days with no response. Another participant did not attend the scheduled interview.

**Table 1.** Inclusion and exclusion criteria.

Inclusion criteria	Exclusion criteria
1. Sonographers employed by the NHS.	1. Sonographers not employed by the NHS.
2. Sonographers who hold a formal ultrasound qualification awarded by the College of Radiographers or a university, including direct entry programmes and apprenticeships.	2. Sonographers who have no formal ultrasound qualification.
3. Sonographers who have been qualified for more than 1 year.	3. Trainee or student sonographers, or sonographers who have been qualified for less than 1 year.
4. More than 50% of their primary healthcare role involves carrying out ultrasound examinations.	4. Less than 50% of their primary healthcare role involves carrying out ultrasound examinations.
5. Sonographers who have never been under any investigation by a professional regulatory body or their employer.	5. Sonographers who have, or are currently, under investigation by a professional regulatory body or their employer.

The interviews took place via Microsoft Teams between July and September 2024. Recordings and transcripts were securely stored on the researcher’s password-protected BCU student OneDrive and were immediately deleted from Microsoft Teams. Once the transcripts had been verified by the participants, the recordings were permanently deleted.

**Figure 2.**
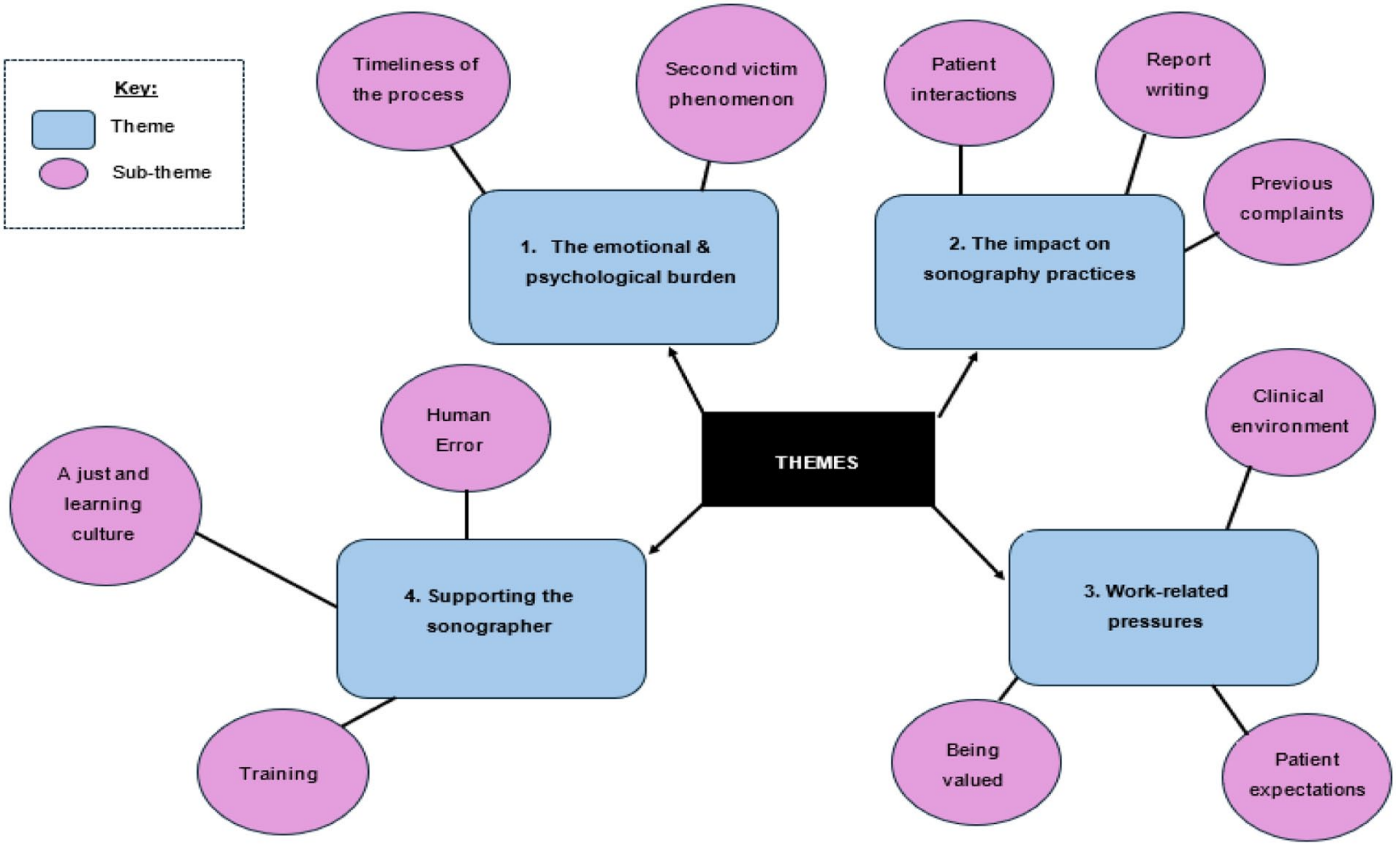
Final thematic map.

Undertaking RTA was a recursive and iterative process, requiring ongoing reflexivity. The researcher first analysed and coded the transcripts, which were eventually refined into four themes with related subthemes that reflected the research aims and questions (Figure 2).

**Figure 3.**
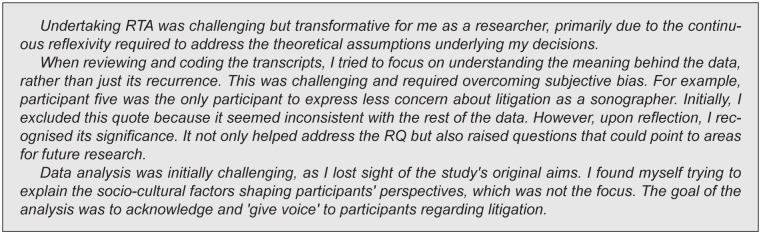
Reflexivity.

## Results

This study included six participants from two NHS Trusts (Table 2). The findings are presented under the four themes generated through RTA. The results are written illustratively, using data extracts to foreground participants’ perspectives, ensuring their voices remained central to the analysis.

**Table 2.** Participant characteristics and interview length.

Participants	Occupation	Gender	Ultrasound experience	Interview length
P1	Direct-entry Sonographer	Female	3 years	17 m 25 s
P2	Radiographer/Sonographer	Female	7 years	26 m 12 s
P3	Radiographer/Sonographer	Female	1 year	24 m 19 s
P4	Radiographer/Sonographer	Female	9 years	41 m 56 s
P5	Midwife Sonographer	Female	14 years	29 m 19 s
P6	Radiographer/Sonographer	Male	9 years	45 m 57 s

### Theme 1: the emotional and psychological burden

Participants described a pervasive fear of litigation, often manifesting as anxiety, sleeplessness, and emotional distress. This fear began early in their careers and, while it sometimes lessened with experience, remained a significant psychological burden:

Our fear is to have that letter in the post saying you’ve done something wrong. (P3)

It was worse when I first qualified. (P2)

However, participants also recognised positive aspects of increased transparency and accountability, noting that professional responsibility to patients’ remains central to their role. The Duty of Candour was viewed as a constructive development, reflecting a shift towards greater openness in healthcare:

. . . the public are much less tolerant of mistakes . . . they are much more informed as to their own rights . . . I think that’s a good thing . . . (P6)

The concept of the SVP was initially unfamiliar, but once explained, all participants identified with its symptoms – guilt, shame, and persistent self-doubt. Although, introspection was also evident:

I wouldn’t be able to sleep at night if I felt like I’d really impacted somebody’s life. (P6)

. . . litigation has a real massive impact on you as a professional. If it didn’t, you would question why it didn’t, because ultimately as healthcare professionals, one of our major components is about caring. (P5)

Although participants empathised with affected patients, they emphasised that the emotional toll on clinicians should not be dismissed. The delay in the litigation process was also identified as exacerbating feelings of emotional harm:

The stress and anxiety that I had was awful. This wasn’t dealt with in a few days. This is something that went on for weeks, if not over a month. I would certainly describe myself as a second victim. (P1)

I think it’s cruel to drag these cases out. It’s not only cruel for the practitioner. It’s cruel for the person going through it. (P5)

Some shared experiences of colleagues withdrawing from certain clinical roles due to the emotional impact of past complaints, reinforcing how litigation fear may influence professional behaviours:

Some of the girls that have done obstetric scans previously, just don’t do them anymore because they’ve had enough of the stress . . . the negative impact it’s had on their lives when things have not gone quite as well. (P6)

### Theme 2: the impact on sonography practices

All participants reported that litigation fear influences their clinical behaviours, often leading to more cautious or defensive practices. Some acknowledged consciously altering how they perform and report scans, while others resisted the term defensive, despite recognising its accuracy:

I am acutely aware of it in that during practice it does affect the way that I perform scans, and it affects the way that I report scans. (P6)

I write things with caution. So yeah, that is being defensive isn’t it to a degree? (P5)

Defensive report writing was a prominent concern. Many felt compelled to use ambiguous language to reduce medico-legal risk, which in turn affected diagnostic confidence:

If medical legal wasn’t an issue, I would be more decisive . . . but you don’t want to put your head on the block. (P6)

Previous complaints were cited as direct catalysts for changes in behaviour, with participants modifying both their clinical documentation and their communication with patients:

This has made me feel particularly closed off towards patients, and very cautious of what I say to them. (P2)

### Theme 3: work-related pressures

Participants highlighted work-related pressures that increase the potential for adverse events (AEs), and thus litigation. Stressors included unrealistic time pressures, particularly from referrers requesting ‘quick scans’, and the overwhelming workload, leading to potential mistakes:

There’s a lot more volume of scans, and if you’re under more pressure, you’re going to make more mistakes. (P4)

Insufficient clinical information is also problematic, requiring sonographers not only to perform the examination but also to gather information that should have already been given:

The lack of information that is often given as to why the patient requires that scan, so then within your time slot you’re trying to get as much information out of your patient as you can while getting them undressed and ready for their scan, performing the scan, and then writing the report. All in that small time slot. And I think that it’s only human nature that rushing is going to make us miss things. (P2)

Sonographers also noted a lack of understanding about the pressures they face, with additional responsibilities, such as mandatory training, supervisory roles, and time constraints adding to the stress:

There are pressures in terms of the amount of mandatory training we’ve got to do, CPD that you’ve got to do, supervising other sonographers, or trainee sonographers. That kind of pressure shouldn’t be discounted, that kind of pressure is very real. (P6)

Finally, participants pointed out the disconnect between patients’ expectations of ultrasound and its actual limitations, which sometimes leads to frustration and increased pressure on sonographers:

They think that ultrasound can give them all the information, all the answers to all different pathologies, but ultrasound has its limitations. (P3)

Although participants frequently emphasised professional accountability, noting that fairness in medico-legal processes includes recognising their responsibilities as autonomous practitioners:

You need to take responsibility for your own training, your own learning . . . your professionalism. (P5)

### Theme 4: supporting the sonographer

Participants discussed challenges surrounding support during litigation processes and training. While sonographers acknowledged the importance of personal accountability in their practice, they also felt that employers should share responsibility, particularly when external pressures contribute to errors:

The employer should hold some accountability as well as the individual, as I find most mistakes are going to happen when there are external pressures. (P3)

Peer support and representation from TUs was recognised as valuable. However, the support from organisational management was seen as lacking, with participants feeling that a blame culture often prevailed despite theoretical promotions of a just culture:

. . . There’s a pointing finger issue and it doesn’t just affect that person that it’s being directed at, it affects the morale of the whole team. (P6)

It was then conveyed that the individual is often having to engage first with management to learn from any potential mistakes:

. . . You’re not aware how you learn from it, or this is how we’ll support you in the future, so you don’t make that same mistake again. It was just an e-mail . . . I had to approach my manager to have this conversation. (P3)

Concerns were raised about the limited time and resources for professional development, with training often only being prioritised after an incident, rather than proactively:

There isn’t always funding available for training staff unless something has gone wrong. It’s like the NHS are always putting out fires, rather than preventing them. (P2)

Some participants also felt that an open learning culture, like that in aviation, could improve support and reduce fear of repercussions:

Being given the time to have chance to discuss it rather than in between patients, or on your lunch break. (P1)

## Discussion

This study explored sonographers’ perspectives of litigation fear and how it influenced emotional well-being or clinical practices. Across the four themes, participants described a substantial burden associated with complaints and AEs, often characterised by anxiety, guilt, and self-doubt.

Litigation concerns shaped day-to-day practice, contributing to defensive scanning and reporting behaviours, while wider organisational pressures intensified the perceived risk of error. Participants also highlighted gaps in organisational support, noting that responses to incidents often felt blame-focused rather than learning-oriented.

Together, these findings illustrate how litigation fear is embedded within both the emotional and structural realities of sonography, shaping professional behaviour and influencing the conditions in which care is delivered.

### The emotional and psychological burden

This study highlights the significant emotional impact that litigation fear has on sonographers, with participants describing anxiety, guilt, and self-doubt that align with features of the SVP.^16^ Although most were unfamiliar with the term, their accounts reflected the psychological strain associated with complaints and AEs. Similar emotional responses have been reported across healthcare professions, where clinicians describe feeling “traumatised” following AEs.^21^ The prolonged nature of investigations intensified this burden, echoing wider concerns that delays in regulatory processes exacerbate distress.^16^

While some participants felt that experience helped them manage their fear, defensive behaviours persisted, suggesting that emotional responses to litigation may become embedded over time. The accounts of colleagues withdrawing from obstetric scanning further illustrate how litigation anxiety can shape career decisions, a trend also noted in previous sonography research.^3^ Given ongoing workforce shortages and rising demand for ultrasound, this pattern warrants attention.

Participants also expressed a strong sense of professional responsibility, balancing empathy for patients with the recognition that clinicians themselves require support. Their reflections suggest that acknowledging SVP does not diminish accountability; rather, it provides a framework for understanding how emotional distress may affect performance and, potentially, patient safety. This aligns with calls for more open discussion of clinicians’ emotional experiences following complaints or AEs.^22^

Some participants also recognised positive aspects of increased transparency, noting that the Duty of Candour supports openness and helps maintain trust with patients. While this shift has contributed to heightened scrutiny, it was also viewed as an important mechanism for promoting honesty and strengthening the patient–practitioner relationship.

### Impact on sonography practices

Fear of litigation influenced how participants approached both scanning and reporting, with many describing more cautious or defensive practices. This included hesitancy in making definitive statements and heightened awareness of how reports might be interpreted. Defensive practice has been documented in other professions, where clinicians report modifying their decision-making to minimise perceived legal risk.^16^ Participants in this study felt that litigation concerns sometimes overshadowed clinical judgement, contributing to over-referral or unnecessary follow-up imaging.

Participants also described modifying their communication with patients after previous complaints, becoming more guarded or withdrawn. Emotional distancing has been identified as a coping mechanism in response to complaints and is associated with burnout and reduced patient engagement.^4^ The findings therefore highlight the need for supportive environments that help clinicians process complaints without resorting to disengagement.

The concerns raised about report phrasing and transparency reflect broader debates within imaging about balancing clarity, accuracy, and sensitivity. Participants’ uncertainty about how patients interpret reports – particularly with increased patient access to results – suggests a need for clearer guidance and potentially more standardised reporting structures. Such measures may help reduce ambiguity while supporting sonographers to communicate confidently and consistently.

### Work-related pressures

Participants described a range of systemic pressures that shaped their daily practice and contributed to the risk of AEs. Increasing workloads, limited appointment times, and staffing shortages were central concerns, reflecting wider pressures reported across the NHS imaging workforce.^23,24^ Their accounts illustrate how rising demand and organisational pressures intersect with litigation fear, creating an environment in which mistakes feel both more likely and more consequential.

A recurring issue was the expectation to accommodate additional or late patients, often framed as a request for a “quick scan”. Participants felt that declining such requests was professionally and socially difficult yet attempting to compress examinations increased the risk of missing clinically important findings. Their reflections highlight a tension between maintaining professional boundaries and responding to the immediate pressures of a busy clinical environment.

Participants also emphasised that the allocated examination time rarely reflected the full scope of the task. Gathering clinical information, preparing patients, performing the scan, and writing the report all had to be completed within tight timeframes. When patients presented with complex needs or limited prior information, these pressures intensified. The mismatch between recommended examination times and real-world demands left participants feeling responsible for risks they could not fully control.

Managing patient expectations added another layer of strain. Participants felt that many patients overestimated the diagnostic capabilities of ultrasound or misunderstood the sonographer’s role, a finding consistent with research showing widespread misconceptions about ultrasound’s purpose and limitations.^25^ These findings suggest a need for clearer communication at referral and during the examination, as well as broader public education about the purpose and limitations of diagnostic ultrasound.

Overall, the accounts in this study show how work-related pressures contribute not only to the potential for error but also to the emotional climate in which sonographers practise. These pressures amplify litigation fear and reinforce defensive behaviours, underscoring the importance of addressing systemic factors alongside individual practice.

Some participants also reflected on the expectations associated with autonomous practice, noting that professional accountability is an inherent part of their role. They recognised that fairness in medico-legal processes includes acknowledging their responsibility for maintaining their own competence and professional standards. This sense of responsibility, while valued, also contributed to the pressures they experienced in an already demanding working environment.

### Supporting the sonographer

Although participants accepted personal accountability for their work, they felt that organisational support during complaints or investigations was limited. Many described experiences that aligned more closely with a culture of blame than with the just and learning culture promoted in policy. Similar concerns have been raised in national reviews, which highlight that investigations often focus on individual error rather than system learning.^26^ The absence of clear feedback, structured debriefing, or visible learning from incidents left participants feeling isolated and uncertain about how to improve future practice.

Peer support and TU representation were consistently valued, yet participants noted that managerial or organisational support was often lacking. Their accounts suggest that support mechanisms are reactive rather than proactive, with learning conversations initiated by individuals rather than embedded within organisational processes. This gap between policy and practice may contribute to the persistence of litigation fear and emotional distress.

Participants also expressed a desire for more formalised support structures, such as professional supervision or protected time for reflection. Supervision has been shown in other health professions to enhance resilience, job satisfaction, and patient care,^1^ yet participants felt that time pressures and service demands made it difficult to access similar support within sonography. Their reflections indicate that creating space for restorative and developmental conversations could help mitigate the emotional impact of complaints and reduce the likelihood of defensive practice.

Taken together, these findings highlight the need for organisational approaches that prioritise learning, transparency, and emotional support. Strengthening these structures may help sonographers feel more confident, reduce the burden of litigation fear, and foster a safer and more supportive working environment.

## Limitations

The sample predominantly comprised white females. Although this reflects the current workforce demographic,^27^ all participants worked in a single UK region, limiting geographical representation. As perspectives may vary across regions, the findings may not be fully transferable. Further research across diverse NHS Trusts is recommended.

## Conclusion

This study demonstrates that litigation fear has a meaningful impact on sonographers’ emotional well-being, shaping both their clinical behaviours and their experience of an already pressured working environment. Concerns about the length and opacity of investigatory processes suggest that more timely and transparent procedures could help reduce stress, particularly for those early in their careers.

Participants described adopting defensive practices, most notably in report writing, which may influence diagnostic confidence and contribute to wider service pressures. Targeted training and clearer guidance in this area could help reduce uncertainty and support more consistent reporting practices.

Workload demands, limited appointment times, and persistent misunderstandings about the sonographer’s role continue to challenge perceptions of safe practice. These findings highlight the value of improved public and professional education, clearer workload expectations, and organisational strategies that acknowledge the realities of current ultrasound services.

A lack of formal support mechanisms and the perception of a blame-focused culture were also evident. While peer support was highly valued, structured professional supervision and a stronger commitment to just culture principles may help mitigate litigation-related stress and promote reflective learning.

Although litigation risk cannot be eliminated, fostering transparency, emotional support, and realistic expectations, both within organisations and among patients, may help reduce its impact and contribute to a more supportive and sustainable working environment for sonographers.
